# Study protocol for the implementation and evaluation of a digital-robotic-based intervention for nurses and patients in a hospital: a quantitative and qualitative triangulation based on the Medical Research Council (MRC) framework for developing and evaluating complex interventions

**DOI:** 10.1186/s12912-022-01088-6

**Published:** 2022-12-09

**Authors:** Christoph Ohneberg, Angelika Warmbein, Nicole Stöbich, Ivanka Rathgeber, Astrid Kruppa, Julian Nast-Kolb, Mattias Felix Träger, Aissam Bahou, Oliver Stahl, Inge Eberl, Uli Fischer

**Affiliations:** 1 Professorship of Nursing Science, Faculty of Social Work, Catholic University of Eichstätt-Ingolstadt, Kapuzinergasse 2, 85072 Eichstätt, Germany; 2 Clinical Nursing Research and Quality Management Unit, University Hospital LMU Munich, Marchioninistr. 15, 81377 Munich, Germany; 3 Cliniserve GmbH, Balanstr. 73/10, 81541 Munich, Germany; 4 Robotise AG, Otto-Hahn-Ring 6, Building 60, 81739 Munich, Germany

**Keywords:** Nursing robotics, Assistive robotics, Digitalization, Technology implementation, Inpatient care, Nursing science

## Abstract

**Background:**

Nurses spend part of their working time on non-nursing tasks. Unnecessary walking distances and the assumption of service activities and other non-care-related tasks take up a lot of space, which reduces the time for direct patient care and demonstrably increases the dissatisfaction of the persons involved. The REsPonSe project aims to relieve nursing staff by using a smartphone app for communication in combination with an autonomous service robot to reduce walking distances and service activities. The technical systems are tested on a nuclear medicine ward and are intended to reduce the radiation exposure of the staff. The aim of this study is to test and evaluate the use and intervention of the technical systems, the acceptance of the users and the change in the utilisation of the nursing service. In addition to findings on usability and manageability, effects on nursing practice, as well as facilitating and inhibiting contextual factors for implementation, will be identified.

**Methods:**

The Medical Research Council (MRC) Framework for Developing and Evaluating for Complex Interventions was chosen as the theoretical basis. The data collection in the Feasibility and Evaluation phase is a triangulation of quantitative and qualitative methods. Standardised observations are planned to collect data on non-care activities and walking distances, and a survey of utilisation by use of a questionnaire based on the NASA TLX. Qualitative individual interviews with patients and group discussions with nursing staff will be conducted. Statements on the subjective experiences, as well as the evaluation of the use of the digital-robotic system in the clinical setting, will be collected. The descriptive evaluation of the usage and retrieval data will provide information on duration, time, requests, and reduced contact times, as well as error and fault messages.

**Discussion:**

The evaluation study will make it possible to represent a variety of perspectives from different interest groups. The results should contribute to the definition of implementation and evaluation criteria and facilitate the integration of digital-robotic assistance systems in nursing acute inpatient settings.

**Trial registration:**

The trial was registered with the German Clinical Trials Register (DRKS) on 16.02.2022: DRKS00028127.

## Contributions to the literature

- The present study is a contribution to a theory-based development and adaptation of digital-robotic assistance systems for nursing settings according to the participatory approach.
- For the evaluation of the complex digital-robotic intervention, a methodological triangulation of quantitative and qualitative methods will be carried out. This enables a more comprehensive description and coverage of the object of research.
- In addition to acceptance, usability and manageability of the technical systems, the object of research also focuses on recording the facilitating and inhibiting contextual factors for subsequent implementation.
- The evaluation of a digital-robotic system in acute clinical care will generate findings on the relief potential of innovative technologies.

## Background

Under the conditions of demographic change and epidemiological developments, it is a challenge for western society to ensure high-quality care that can be financed in the long term [1]. Under this demand, the field of nursing is confronted with various challenges, such as a shortage of skilled workers. This has a direct impact on the professional field. Nurses spend a considerable part of their working time on tasks that are not originally nursing tasks. Unnecessary walking, service activities and other non-nursing interventions take up a lot of space, reducing the time available for direct patient care and demonstrably increasing dissatisfaction [2, 3]. Furthermore, requests from colleagues and patients often interrupt nursing tasks, as well as the call system or telephone calls do. These interruptions are additional stressors and can reduce the quality of nursing care as well as the effectiveness of work processes, which leads to an additional burden in the daily work of nurses [4, 5].

Robotic systems and digital interventions have the potential to relieve nursing staff in acute and long-term inpatient care as well as in outpatient care, but also to take over general support tasks in the home care of people in need of care. They should support nursing professionals and improve the quality of life of patients without compromising the quality of care [6, 7]. Service and assistance robots should provide a possibility of relief. According to Mallouf et al. [8], the term service robotics includes technical systems that support humans in performing services and activities in a partially or fully automated way. They are used in non-industrial fields of application and are operated by persons who are not specially trained. In addition to informational and sensory functions, service robotics are able to autonomously reach the destination position and perform complex tasks consisting of several steps and materials [9, 10].

### Implementation of robotic systems in nursing practice

The use of robotic assistance systems in the field of nursing includes various challenges: These relate to technological, clinical, financial, insurance, psychological, social, ethical and legal issues. Technological challenges include indoor navigation, manipulation, IT-security, telecommunication and the integration of robots with existing technologies in the hospital. To ensure indoor navigation, corridors need to be kept clear of obstacles. On a user-centred level, the perception and attitude of users towards robotic systems in care plays a key role for future technological developments. This applies both to the perspective of patients and older people, as well as to that of informal and professional caregivers. If the technical systems interfere with the social dynamics of a group or require additional effort, this can affect acceptance and lead to negative behaviour [11–13].

A systematic literature review shows that current studies have focused in particular on improving the usability and manageability of robotic systems. In addition, the majority of robotic systems are still in the development and testing phases [14].

The present study makes a theory-based contribution to the development of implementation and evaluation criteria for technological innovations and explores initial effects on nursing care in the acute clinical setting.

### Aim

This evaluation study is a part of the ResPonSe project (Robot system to relieve the nursing staff of service activities). The aim of this study is to test and evaluate the use and intervention of the technical systems, the acceptance of the users and the change in the utilisation of the nursing service. In addition to findings on the usability and manageability of the digital-robotic assistance system, effects on nursing practice, as well as facilitating and inhibiting contextual factors for implementation, will be determined. The evaluation study thus provides a contribution to the structured, participatory, and scientifically accompanied integration of a digital-robotic assistance system in acute inpatient settings.

## Methods/Design

The theoretical basis for this study is the Medical Research Council (MRC) framework for developing and evaluating for complex interventions. Data collection will take place in the Feasibility and Evaluation phase [15] as a triangulation of quantitative and qualitative methods [16] (Table 1). According to Skivington et al. [15], a complex intervention can be considered complex because of the characteristics of the intervention itself. Such characteristics include, for example, the required expertise and skills of those implementing and using the intervention, the number of components or users involved, and the setting. Here, the focus is on implementation criteria, assessing the feasibility of the intervention and its usefulness and acceptability. The evaluative focus takes up a context-related understanding. Thus, changes brought by the digital-robotic assistance system in acute inpatient nursing care will become the subject of research.

The triangulation of quantitative and qualitative methods was chosen to enable a complementation of perspectives and a more comprehensive description, recording, and explanation of the subject area [17]. The chosen triangulation of methods is intended to mutually complement the research findings [18]. Integration in the research process takes place at the time of data collection and analysis.

### Project REsPonSe

The evaluation study is being conducted as part of the REsPonSe project (Robot system to relieve the nursing staff of service activities). In the project, a service robot and a smartphone application are linked and adapted to the conditions of an acute hospital. The aim of the project is to relieve nursing and service staff of some service activities by using the smartphone app for communication in combination with an autonomous service robot. It is expected that the use of the technologies will also reduce the contact and thus the radiation exposure for the nursing staff. The pilot phase will start in April 2022 and is planned for six months.

### Intervention

By using a communication app, patients are able to requests to the nursing staff who can accept the requests or forward them to a colleague. Further information are exchanged via a chat function. Simple service requests, such as deliveries of drinks or cool packs, are forwarded directly to the service robot via the app. The robot autonomously delivers the ordered item to the patient's room. After washing and disinfecting their hands, the person making the request can receive the ordered item at the door of the room. During the whole pilot phase, the service robot will only be in operation when trained personnel from the development company are present. This allows for immediate intervention in case of technical problems. The communication app does not replace the emergency bell. In the case of urgent emergency patient transports, after getting the emergency signal from the app, the robot will autonomously move to the closest defined saftey spot on the ward or can be manually pushed out of the way via a safety button.

### Setting

The study will be conducted in a nuclear medicine ward at a German maximum care hospital. There are 16 beds available for therapy with open radionuclides. The length of stay of the patients depends on the amount of radioactive substance used and is about four to seven days. Due to the radiation exposure caused by the therapy, the patients usually have to spend 2–3 days isolated in their room. After the radiation level has fallen below a critical value, the patients can move around the ward or the ward's own terrace without any restrictions.

### Sample and inclusion criteria

The following inclusion criteria were defined for participation in the evaluation study:

- Nursing staff of the pilot ward who are involved in the testing of the combined digital-robotic assistance system with informed consent.
- Service staff of the pilot ward who are involved in the testing of the combined digital-robotic assistance system with informed consent.
- The patients of the pilot station who are capable of giving consent and who are involved in the testing of the combined digital-robotic assistance system with informed consent.

Patients who are under care or unable to give informed consent are excluded.

Information and recruitment of participants will take place via information and training events, information flyers, ward management and personal contact with the researchers. The surveys of the hospital staff will only take place after approval by the staff representatives. The participants do not derive any benefit from their participation. There is no remuneration or reimbursement of expenses.

### Data collection methods

The data collection contains three sub-surveys (Table 1) and will be conducted between April and October 2022.

**Table 1:** Study design

	**Aim**	**Design**	**Participants**	**Estimated sample size**
**Evaluation of nursing tasks, distances walked and utilization in nursing services**	Description of the distances travelled and the assumption of activities that are not related to care, as well as a survey of the utilisation of these activities	Standardized observation and questionnaire	Nurses	30 observations and 100 questionnaires
**User perspective on the usefulness, useability and integration of technologies**	To investigate the perspective of the users with regard to the integration of the robotic system and its usefulness and effect on nursing care	qualitative cross-sectional survey	Nurses, Patients, Service assistants	Group discussion: Approx. *n* = 5; Individual Interviews: data saturation
**Data evaluation of the technologies**	Use behaviour of robot and app	quantitative cross-sectional survey	Robot, Application	Approx *n* = 200–300

### Evaluation of nursing tasks, distances walked, and utilization in nursing services

The aim of this sub-survey is to describe the walking distances covered and the assumption of non-care activities, as well as to record the use of these activities at several points in time. For this purpose, standardised, non-participatory observations [19] will be carried out by the members of the clinical study team. The following data will be collected: request number, requesting person, category of activity (non-routine service or care), duration and interruptions, walking distances (short =  < 10 m, medium length = 10 < x < 30 m; long =  > 30 m). A questionnaire based on the NASA Raw TLX (Task Load Index) [20–23] will be administered to all nurses and ward assistants at the end of each observed shift to collect data on utilisation. The questionnaire uses a 20-point Likert scale (from "very low" to "very high") to record the nurses' subjective assessments of interruptions, complexity, situational stress, performance, effort, frustration and comparability with other shifts.

#### Sample size

Data collection will take place during five data collection periods: before the introduction of the technologies at the pilot station, during the introduction of the smartphone app, during the introduction of the robot, the first period after the introduction of both technologies and finally during routine use. A total of six observations will be carried out during early and late shifts at each period of data collection. One nurse will be accompanied for the entire shift during an observation unit.

#### Data analysis

The observation data will be categorised according to Schnell et al. [24], entered into an Excel database and sorted by type of activity. A statistical-descriptive evaluation of the data is planned.

### User perspective on the usefulness, usability, and integration of technologies

The aim of the qualitative research part is to examine the perspective of the users (nursing staff, service staff, patients) with regard to the integration of the digital-robotic assistance system and its usefulness and effects on nursing care. For this purpose, episodic individual interviews are conducted in addition to group discussions. These enable not only an experience-based, but also a defining and explanatory, view of the research object [25, 26]. For both the individual and group discussions, the interview guide includes the following topics:

- Technology acceptance, impact on care and work processes;
- Perceived usefulness and usability;
- Facilitating and inhibiting contextual factors for integration in an acute hospital.

The patients will be interviewed in the form of individual interviews. It is also planned to conduct two group discussions with at least four nurses, one at the beginning and one at the end of the pilot phase. The first group discussion will focus on initial experiences with the technologies and their benefits and effects in everyday professional life. The focus of the second group discussion will be on the perceived changes during the deployment period and the integration of the digital-robotic assistance system into the acute inpatient setting. This methodological approach is intended to create the opportunity to report different experiences in mutual exchange and to discuss the development of robotic technologies in nursing [16, 27]. For the sample description, age, gender and occupational status are recorded [28].

#### Sample Size

The number of interviews depends on data saturation. Further interviews will not be conducted if it is determined during the analysis that no new insights will be gained through further interviews [29].

#### Data analysis

The audio files of the individual and group interviews will be transcribed according to the rules of Dresing & Pehl [30]. The individual interviews will be analysed using qualitative content analysis according to Kuckartz [31], and the documentary method according to Bohnsack [32, 33] will be used to analyse the group discussions. The data analysis will be computer-aided with the software MAXQDA 2022.

### Data evaluation of the technologies

A quantitative cross-sectional survey [34] will be used to find out whether the digital robotic assistant system can be integrated into the processes of a general ward in the acute inpatient area and to which extent nursing staff and patients use the technologies. The following parameters will be collected anonymously during the piloting (Table 2).

**Table 2:** Parameters of the data evaluation of the technologies

**Smartphone Application**	
** Day**	Date of the day of use
** Time**	Request daytime
** Request type**	Selected request in the app
** User**	Requesting Person: Patient, Nurse, Service assistant
** Operator**	Nurse, Service assistant, Robot
** Success**	performed sucessfully: yes, no
**Robot**	
** Duration**	Duration of the operation in minutes
** Route**	Distance covered in metres

#### Sample size

The sample includes all requests made via the app during the pilot.

#### Data analysis

In addition to the statistical-descriptive evaluation of the individual parameters (mean, standard deviation, median, mode, 1st and 3rd quartile, interquartile range), relative and absolute frequencies will be calculated according to the data. Furthermore, an explorative, hypothesis-generating evaluation strategy will be pursued. Based on the distribution of the data as well as the scale levels, explorative correlation measures will be calculated using the Wilcoxon test and the correlation coefficients. If significant correlations are demonstrated, linear regression analyses will be considered [34]. Data management and analysis will be carried out with the statistical software IBM SPSS Statistics, version 25.

### Data security and processing

The consent of the responsible data protection officer of the clinic was obtained for the entire project on the basis of Art. 6 of the General Data Protection Regulation (DSGVO) (1042a., 12.01.2021).

During data collection and data evaluation, the data are pseudonymised. Each participating person receives a three-digit pseudonymised identification number. If personal statements or statements that can be traced back to individuals are given in the context of the qualitative data collection, these will be anonymised and treated as strictly confidential. The audio files will be deleted after the end of the data analysis. The participants will be informed about data protection by an information leaflet. The data will not be passed on or transferred to third parties. The data, as well as their access, are secured by the network of the research institutions.

The standardised observations will be transferred to an Excel file. Following this, the activities will be summarised in categories. The interviews will be audio-recorded with a recording device and transcribed word-for-word [35].

All data analysis will be carried out in such a way that at the end of the data processing, no trace back to the participants can be created. Furthermore, data processing and storage will take place on encrypted computers to which only members of the research team have access.

### Ethical considerations

Before the beginning of the study, the responsible ethics committee granted ethical approval for the study (project number 21–1202). The study will be conducted in accordance with the 2013 version of the Declaration of Helsinki [36]. Within the framework of free and informed consent, the participants will be informed in detail in advance, both verbally and in writing, about the project and the aims of the study parts [37]. Informed consent will be obtained from all participants prior to commencement of the study. This can be cancelled at any time without giving reasons.

### Dissemination

The results of the study will be made available to the public in the form of congress and journal publications as well as through the project partners' homepages.

## Discussion

In addition to challenges for the implementation of digital robotic systems, methodological approaches and existing evidence on the topic will be discussed.

### Implementation challenges

Kirschling et al. [12] described spatial requirements, such as keeping corridors clear of obstacles, as a challenge when implementing robotic assistance systems in an acute inpatient setting. Before the technical systems can be used on the pilot station, the internal clinical requirements, e.g., fire protection, work safety and hygiene, must be met. For example, patients must wash and disinfect their hands before coming into contact with the robot in order to exclude potential transmission of pathogens or radioactive substances. In addition, the technical use requires further conditions such as a stable WLAN network as well as spatial requirements such as non-reflective floor or wall materials. These could cause interference with robotic sensors.

The design of organisational interfaces and the unequal distribution of the benefits of technical systems among the members of an organisation are also described as challenges in the literature. While the applications are designed to provide a collective benefit to the organisation, they first require more work from some members of the organisation, which can lead to a rejection of these systems. If the robotic system interferes with the social dynamics of a group, it can also lead to rejectionist behaviour [13]. The question of acceptance is therefore related to the benefits and the use of the technical systems. In addition to integrating these aspects into the qualitative research part, project participants need to understand the needs of the users in the ongoing project and adapt or change application possibilities in an agile way during the pilot phase.

### Available evidence and methodological discussion

In a systematic literature search on assistive robotics in care, studies with different foci were found. One area deals with the attitude and acceptance of care professionals and people in need of care towards robotic systems for care. The results are heterogeneous: on the one hand, care professionals and people in need of care were open to robotic systems. On the other hand, technologies have been seen as having the potential to contribute to stigmatisation and dehumanisation [38, 39]. In addition to the uncertainty in dealing with robotic systems [38], the technical complexity of the systems and the lack of user participation in development and implementation processes also have an impact on acceptance [40]. The attitude and acceptance towards robotic systems in particular was researched with qualitative methods. Different interview forms, such as semi-structured individual interviews or group interviews, were conducted [39–41]. Researching these aspects using qualitative methods is considered to be purposeful.

Feasibility and evaluation studies have focused on the issue of improving the usability and manageability of robotic systems at different stages of development and in different settings [12, 38, 42–45]. Quantitative research approaches [12, 43, 45] or studies with a mixed methods approach [38, 42, 44] have been pursued for this purpose. Quantitative approaches evaluated, among other things, technical usage of the technologies or used questionnaires to survey satisfaction, usefulness or user experience [12, 43, 45]. The evaluation of the usage and retrieval data enables a quantifiable representation of the actual use of the systems within the test period. Through the method of standardised observation and the questionnaire on workload, an approach was put forward for discussion that concentrates specifically on statements on relief in nursing practice.

The theory-led and participatory development and testing of a digital-robotic system based on the MRC framework [15] can be considered positive at this point in time.

### Limitations

The use of the digital-robotic assistance system is limited to the phase of piloting on a general ward with a very specialised field. The majority of patients are mobile and require minor assistance. In addition, there are possible technical problems or adaptations that can affect the acceptance behaviour of the users (patients, nursing staff, service personnel).

There is a risk of selection bias [46] in the selection of interviews with patients, as it is assumed that patients who are positively disposed towards the technologies are more willing to be interviewed. In the nurses' group discussions, group dynamics and status quo bias [47] could determine statements and influence the outcome. During standardised observations, there is a risk of selection and performance bias [46, 48]. In principle, the risk of social desirability must be taken into account in all study components.

### Prospect

International studies show that the development of service and assistance robotic systems is still in its early stages, but is progressing steadily. Various pilot projects are being tested in experimental phases together with user groups. This is mainly about usability, development and application procedures. However, the integration into a nursing setting has hardly gone beyond the test phase and the development of assistive and service-oriented robotics has hardly taken into account the view of the users, e.g., nursing professionals [13, 49]. Practitioners, nurses and engineers must work together in an interprofessional way and integrate people in need of care and their relatives in a participatory way in the development and testing phases.

On the part of the researchers, the effects on nursing practice, as well as promoting and inhibiting contextual factors for implementation, must be ascertained [50]. In addition to developing implementation and evaluation criteria, models such as the MRC Framework for developing and evaluating complex interventions need to be further developed and adapted to the requirements of technology development in nursing practice. As a first suggestion, the participatory role of potential users should be emphasised more strongly in scientific models. Furthermore, the context and the associated challenges for development and implementation must be taken into account. In parallel to the participation of nurses, patients and relatives, practice and technology developers must work closely together in an agile manner.

## Data Availability

The datasets generated or analyzed during the current study are not publicly available due to incompletion but will be available from the study directors on reasonable request. Data containing personal patient information cannot be provided due to data protection.

## Abbreviations

DSGVO: Datenschutz-Grundverordnung
LMU: Ludwig-Maximilans-University Munich
MRC: Medical Research Council
NASA TLX: NASA Task Load Index
REsPonSe: Robot system to relieve the nursing staff of service activities
